# Isolation of an *ABA Transporter-Like 1* Gene from *Arachis hypogaea* That Affects ABA Import and Reduces ABA Sensitivity in *Arabidopsis*

**DOI:** 10.3389/fpls.2017.01150

**Published:** 2017-06-30

**Authors:** Kui Ge, Xing Liu, Xiaoyun Li, Bo Hu, Ling Li

**Affiliations:** Guangdong Provincial Key Laboratory of Biotechnology for Plant Development, College of Life Sciences, South China Normal UniversityGuangzhou, China

**Keywords:** peanut, AhATL1, ABA transporter, AtABCG40, stomatal movement, drought stress

## Abstract

Abscisic acid (ABA) transporters are essential for the transport of ABA from its sites of synthesis to its multiple sites of action within plants and are key players in plant stress responses. Despite their importance, there is limited information on ABA transporters in crop plants. In this study, we isolated and characterized an *ABA transporter-like 1* (*AhATL1*) gene from peanut (*Arachis hypogaea* L.) whose cognate protein, AhATL1, is a member of the ATP-binding cassette transporter G subfamily and localizes to the plasma membrane. The expression of both the *AhATL1* transcript and the corresponding protein were upregulated by water stress and treatment with exogenous ABA. Overexpression of *AhATL1* in ecotype Columbia (Col) Arabidopsis (*AhATL1-OX*) plants reduced ABA sensitivity. When *AhATL1-OX* and Arabidopsis Col plants were subjected to dehydration stress, the expression of *9-cis-epoxycarotenoid dioxygenase 3* (*AtNCED3*) and *responsive to desiccation 29 A* (*AtRD29A*) accumulated rapidly in rosette leaves of both lines. In contrast, while expression of *ATP-binding cassette G 40* (*AtABCG40*) was increased in Col rosette leaves, there was no change in expression of *AtABCG40* in *AhATL1-OX* leaves. Similarly, water loss from detached leaves of *AhATL1-OX* plants was more rapid than from Col leaves. Therefore, we suggest that the function of *AhATL1* is probably to modulate ABA sensitivity by specifically influencing ABA import into cells.

## Introduction

Abscisic acid (ABA) is a key phytohormone involved in a host of biological processes, including embryo and seed maturation, postgerminative growth, and abiotic stress responses (Tuteja, 2007). Drought is the most damaging abiotic stress affecting plant productivity (Govind et al., 2009). When higher plants are exposed to drought, the levels of endogenous ABA increase and downstream signaling pathways are activated (Hwang et al., 2016). Indeed, many drought-responsive genes are induced by exogenous application of ABA (Li T. et al., 2016; Zhao et al., 2016; Li et al., 2017). Local active ABA levels reflect a balance of ABA biosynthesis and inactivation by turnover or conjugation, and levels are further modified by compartmentation and transport (Nambara and Marion-Poll, 2005; Finkelstein, 2013). The translocation of ABA between cells, tissues and organs also plays an important role in the physiological response of plants to stress conditions. For example, ABA is rapidly synthesized in root vascular parenchyma under root stress, and is then rapidly transported to leaf tissues, where it induces stomatal closure in peanut and Arabidopsis (Boursiac et al., 2013; Puértolas et al., 2015; Hu et al., 2016).

ATP-binding cassette transporters are a highly conserved family of ATP-driven pump proteins consisting of two hydrophobic transmembrane domains (TMDs), which constitute the membrane-spanning pore, and two cytosolic domains, known as the nucleotide-binding domains (NBDs) or nucleotide-binding folds (NBFs), which contain the ATP-binding Walker A and B motifs (Kerr et al., 2011). In Arabidopsis, four ABA transporters have been identified (AtABCG25, AtABCG40, AtABCG30, and AtABCG31), all four of which are ATP-binding cassette transporter G subfamily members (Kang et al., 2010, 2015; Kuromori et al., 2010; Kanno et al., 2012). AtABCG25 is involved in exporting ABA from the vasculature (Kuromori et al., 2010), while AtABCG40 is a plasma-membrane ABA-uptake transporter in guard cells, and is necessary for timely closure of stomata in response to drought stress and seed germination (Kang et al., 2010, 2015). AtABCG30 mediates ABA-uptake into the embryo, while AtABCG31 brings about ABA secretion from the endosperm (Kang et al., 2015). These data indicate that the ABA transport system plays a significant role in water deficit tolerance and growth regulation. Therefore, investigation of the function of ABA transporters in ABA signaling is essential for an understanding of plant resistance to water stress. However, ABA transporters have not yet been characterized in crop plants of economic importance.

Like other crops, the peanut plant (*Arachis hypogaea* L.), which is a major oil and protein seed crop worldwide, is subjected to periodic soil moisture-deficits of varying degree and duration that can result in substantial loss of yield (Nautiyal et al., 2002). Therefore, how water stress affects growth sensitivity and stomatal movement in peanut, and the role of ABA biosynthesis and transport in this stress response, is of considerable importance. In our previous study in peanut, we found that ABA was rapidly synthesized in roots treated with polyethylene glycol 6000 (PEG 6000), mainly accumulating in root vascular parenchyma, and was then quickly transported to the leaf tissues where it induced stomatal closure (Hu et al., 2016). However, it is currently unclear how ABA transport to leaves from biosynthesis sites affects stomatal movement during water stress in peanut.

Here we report an *ABA transporter-like1* (*AhATL1*) gene from *A. hypogaea* that encodes a plasma membrane-localized protein containing ABC family domains. *AhATL1* gene transcription and protein expression are enhanced when peanut roots are treated with PEG 6000 or exogenous ABA. Furthermore, no AhATL1 protein is detected when peanut root is incubated with ABA biosynthesis inhibitor fluridone (Flu) following PEG 6000 treatment. On the other hand, AhATL1 protein expression is similar to that with PEG 6000 treatment alone if plants are pretreated with Flu and then Flu is replaced with ABA. This suggests that AhATL1 expression specifically responds to drought stress via ABA signals in peanut. Overexpression of *AhATL1* in Arabidopsis Col lines enhances seed germination, and also increases the axial root length compared with Col plants after exposure to various concentrations of ABA, indicating that *AhATL1* decreases ABA sensitivity when expressed heterogenously in Arabidopsis. At the same time, while drought increased *AtABCG40* expression in Col plants, there was no such change in *AhATL1-OX* plants. Moreover, stomatal movement in *AhATL1-OX* plants in response to drought stress was lower than that in Col plants, while water loss rates were higher. In summary, *AhATL1* appears to reduce ABA sensitivity by specifically influencing ABA import.

## Materials and Methods

### Peanut Plant Material and Growth Conditions

Peanut (*Arachis hypogaea* L.) cultivar Yueyou 7, a line resistant to water stress, was provided by the Crop Research Institute, Guangdong Academy of Agricultural Sciences, China. Seeds were soaked in water for 12 h, and then placed on moist filter paper in a growth chamber with a cycle of 16 h light from fluorescent and incandescent lamps (200 μmol m^-2^ s^-1^) at 26°C followed by 8 h darkness at 22°C for 48 h until the cusp was exposed. Then germinated seeds were sown in saturated peat-containing soil in a growth chamber. Plants were well watered as previously (Su et al., 2015).

### Isolation and Sequence Analysis of *AhATL1* from *Arachis hypogaea* L.

First-strand cDNA was synthesized by reverse transcription (RT) of 1 μg total RNA from peanut leaves, using 200 units Superscript III Reverse Transcriptase (Invitrogen, catalog no. 18080) and 500 ng oligo-dT primer under the following conditions: 70°C for 10 min, 42°C for 1 h, and 15 min at 70°C (Su et al., 2015). The *AhATL1* open reading frame was amplified by PCR using the following primers: *AhATL1* forward primer 5′-ATGGCATCTGAAACCATGGTGGT-3′, *AhATL1* reverse primer 5′-TTATGCTTGATGACTTAGTGCAG-3′. PCR amplification was performed as follows: 94°C for 5 min, then 35 cycles of 94°C for 30 s, 55°C for 3 min and 72°C for 1 min, with a final extension step at 72°C for 10 min. PCR products were ligated into the pMD19 T-vector (TaKaRa, catalog no. 6013) and confirmed by sequencing. The AhATL1 amino acid sequence and transmembrane structure was analyzed, respectively, by BLAST^1^ and TMHMM 2.0^2^.

### Peanut Plant Treatments

To simulate the effects of different plant hormone treatments, peanut plants were gently removed from soil at the four-leaf seedling stage, maintained in 1/8 Murashige and Skoog medium (MS) for up to 2 h, and then replaced with 100 μM ABA, 100 μM gibberellin_3_ (GA_3_), 50 nM 1-naphthylacetic acid (NAA) or 25 μM 6-benzylaminopurine (6-BA) for 1, 3, 5 or 8 h. For pretreatment with the ABA biosynthesis inhibitor fluridone (Flu), the peanut taproot was first pretreated with 100 μM Flu for 2 h followed by treatment with 20% (w/v) PEG 6000 solution or 100 μM ABA for 1, 3, 5, 8, 12 or 24 h. To examine the effects of water stress on peanut plants, taproots were transferred to 1/8 MS for 2 h and then replaced with 20% (w/v) PEG 6000 solution for 1, 3, 5, 8, 12 or 24 h under standard growth conditions.

### Protein Extraction

Peanut leaf samples (first functional leaves; 100 mg) were frozen in liquid nitrogen immediately after treatment and stored at -70°C until further use. Total leaf protein samples were extracted by grinding leaves in liquid nitrogen and 1 mL lysis buffer [50 mM Tris-HCl pH 7.2, 10% glycerol, 2% SDS, 1% β-mercaptoethanol, protease inhibitors cocktail (Roche), 100 mM PMSF (Sigma)].

### Antibody Preparation and Immunoblotting

To analyze the production of AhATL1 protein in peanut leaf samples by immunoblotting, three synthetic peptides (each of 12–14 amino acid residues) corresponding to residues 69–82 (QKPSDETRSTEERT), 132–143 (GKITKQTLKRTG), and 328–340 (GVTEREKPNVRQT) of the AhATL1 protein were used to prepare three polyclonal anti-AhATL1 antibodies in rabbits. Total protein from peanut leaves was used to test antibody specificity.

The concentration of protein samples was determined using a TaKaRa BCA Protein Assay Kit (TaKaRa, Code No. T9300A). Total leaf protein was suspended in 5 × SDS–PAGE loading buffer (0.25 M Tris-HCl, pH 6.8, 10% SDS, 50% glycerol, 5% 2-mercaptoethanol), then loaded and run on 15% polyacrylamide gels, which were subsequently stained with Coomassie Brilliant Blue or blotted onto a 0.22 μm PVDF membrane. The membrane was blocked overnight in Tris-buffered saline with 0.1% Tween 20 (TBST, pH 7.6) containing 5% dry milk and then incubated with 0.01–0.05 mg/mL of anti-AhATL1 antibody for 14 h at 4°C. After washing with TBST three times, the primary antibody was detected with secondary HRP-labeled goat anti-rabbit IgG (H + L) antibody (Dingguo, catalog no. HD001-1) at room temperature for 45 min. Visualization was achieved using the ECL system (Millipore, catalog no. 345818).

### Expression Analysis

Total RNA was extracted from peanut leaf or *Arabidopsis thaliana* rosette leaf tissue after various treatments using a TRIzol Kit (TaKaRa), and a Prime Script TM RT Reagent Kit (Perfect Real Time, TaKaRa) was used for RT. Real-time quantitative PCR (qRT-PCR) was performed with the ChamQ SYBR qPCR Master Mix (Low ROX Premixed, Vazyme Biotech Co., Ltd) in an ABI 7500 system to quantify *AhATL1* transcript levels. Each reaction consisted of 10 ng cDNA, 0.05 mM primers and 5 μL 2 × ChamQ SYBR qPCR Master Mix in a final volume of 10 μL. The reactive cycle consisted of 95°C for 30 s, then 40 cycles of 95°C for 5 s and 60°C for 34 s. Gene expression level was calculated using the relative 2^-ΔΔCT^ method (Li et al., 2013). Expression data for *A. hypogaea* and *A. thaliana* were normalized using the geometric mean (geomean) of the validated housekeeping genes *AhACTIN* and *AtACTIN2*, respectively, as described (Su et al., 2015; Li X. et al., 2016). Primers are listed in Supplementary Table S1.

### Subcellular Localization

Full-length *AhATL1* cDNA was amplified using KOD FX polymerase (Toyobo) with forward primer 5′-CGGGTTC GAAATCGATGGATCCATGGCATCTGAAACCATGGTGGT-3′ and reverse primer 5′-GTCCTAGGCTACGTAGGATCCTTATGCTTGATGACTTAGTGCAG-3′. PCR products were first cloned into pMD19 and confirmed by sequencing. Then, the *AhATL1* open reading frame was transferred into the plasmid *p35S::eGFP*, which contains the *35S* promoter, to obtain *p35S::AhATL1-eGFP*. Constructs were then transformed into *Arabidopsis* protoplasts, with *p35S::eGFP* as a positive control. After growing in darkness for 16 h, GFP fluorescence was determined by confocal microscopy (Carl Zeiss LSM 710).

### Overexpressing Arabidopsis Plants

The *p35S::AhATL1-eGFP* construct was transformed into Arabidopsis Col plants by the floral dip method using *Agrobacterium tumefaciens* strain *EHA105*. From T2 plants, transgenic lines were identified by PCR. T3 seeds were used for subsequent experiments. Plants were grown on peat-containing soil with a daily cycle of 16 h light and 8 h dark in well-watered conditions at 22 ± 2°C and 60–70% relative humidity.

### Arabidopsis Seed Germination, Cotyledon Greening and Root Growth

*AhATL1-OX* and Col seeds were used to assess the effects of exogenous ABA at different concentrations on seed germination, cotyledon greening and root growth. Seeds were surface sterilized in 70% ethanol containing 0.5% Tween-100 and were air-dried on filter paper. To examine the effects of ABA on germination and the development of green cotyledons, seeds were sown on 1/2 MS medium with 0.8% agar containing 0, 0.1, 0.5 or 2 μM ABA. After 2 days stratification at 4°C, germinating seeds were transferred to a growth chamber with a daily cycle of 16 h light and 8 h dark at 20 ± 2°C. Germination was observed and recorded every 6 h until 96 h. Three days after germination on 1/2 MS medium, seedlings were transferred to new 1/2 MS medium with 0.8% agar and 2% sucrose containing 0, 10 or 50 μM ABA for root growth experiments. Root growth was recorded by photography 7 days after transfer to the new plates and measured using Digimizer software (Li X. et al., 2016).

### Stomatal Movement Assay

Detached rosette leaves from 3-week-old *AhATL1-OX* and Col plants were used to determine stomatal apertures. For assays of stomatal movement under normal conditions or following dehydration treatment, detached whole leaves were floated on “open stomatal buffer” containing 10 mM MES, 5 mM KCl, 50 mM CaCl_2_, pH 6.15, in a growth chamber under incandescent lamps (200 μmol m^-2^ s^-1^) at 20°C for 3 h to allow stomata to open, and were then transferred onto filter paper in a growth chamber under incandescent lamps (200 μmol m^-2^ s^-1^) at 20°C for 2 h for drought treatment. Stomatal apertures were measured as described previously (Kang et al., 2010). Digimizer software was used to measure stomatal apertures.

### Statistical Analysis

Quantitative data were expressed as mean ± SD. The statistical significance of experimental data was assessed by Student *t*-test or ANOVA (one-way analysis of variance with a LSD *post hoc* test), as appropriate, using the SPSS17.0 statistical package.

### Accession Numbers

Sequence data from this article can be found in the Arabidopsis genome initiative database or National Center for Biotechnology Information under the following accession numbers: *AtNCED3* (AT3G14440), *AtABCG40* (AT1G15520), *AtABCG25* (AT1G71960), *ATACT2* (AT3G18780), *AtRD29A* (AT5G52310), *AhATL1* (KY621345), *GsABCG11* (KHN26506), *MnABCG11* (XP_010094142), *AtABCG11* (NC_00370), and *MtWBC* (XP_010094142).

## Results

### *AhATL1* Belongs to the ABCG Subfamily

**FIGURE 1:**
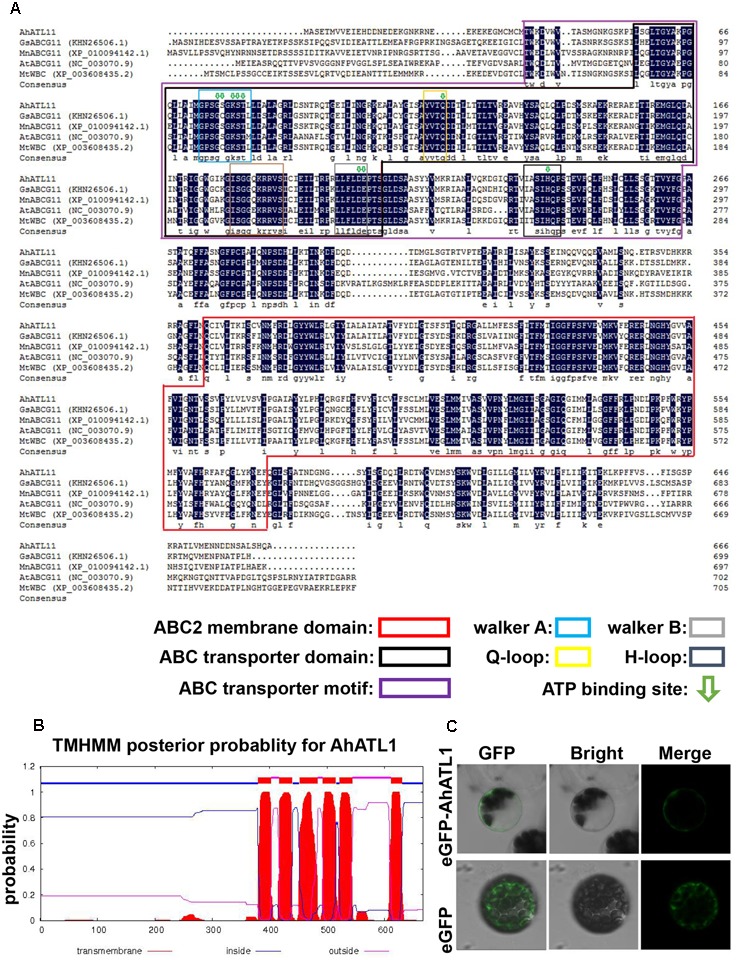
AhATL1 is a member of the ABC transporter family. **(A)** Alignment of amino acid sequences of AhATL1 with four ABCG subfamily proteins. AhATL1: GenBank number KY621345; GsABCG11: GenBank number KHN26506.1; MnABCG11: GenBank number XP_010094142.1; AtABCG11: GenBank number NC_00370.9; MtWBC: GenBank number XP_010094142.1. Identical amino acid residues are shaded in dark blue. Conserved functional domains are marked with rectangular boxes of different colors, as indicated in the key. ATP-binding sites are labeled with green hollow arrows. **(B)** AhATL1 protein contains a total of six transmembrane structures. Transmembrane helical structures are represented by amino acid residues 380–402, 417–439, 452–483, 493–515, 522–544, and 612–630 of the AhATL1 protein. Consequently, residues 1–379, 440–451, 516–521, and 631–667 are located inside the bilayer (i.e., are intracellular). Amino acid residues 403–416, 484–492, and 545–611 are thus outside the bilayer. AhATL1 transmembrane structure was analyzed using TMHMM 2.0 (online at http://www.cbs.dtu.dk/services/). **(C)** The intracellular localization of AhATL1 in Arabidopsis protoplasts. Expression of the fusion protein AhATL-eGFP or eGFP alone after transient expression in protoplasts, determined by confocal and bright-field microscopy.

We conducted a BLAST alignment to find conserved protein domains in AhATL1 (Gen Bank accession no. KY621345) after translation of the *AhATL1* open reading frame. Two domains were found that characterize ABC transporters: an ABC2 membrane domain (residues 366–572) and an ABC transporter domain (residues 56–207). Sequences signifying Walker A, Walker B, Q-loop, D-loop, and ATP-binding sites were also found in the AhATL1 protein sequence (**Figure 1A**). A prediction of secondary structure suggested the protein has six transmembrane structures (**Figure 1B**), consistent with AhATL1 being a member of the ABCG family (Kerr et al., 2011). To determine the subcellular localization of AhATL1, we engineered a plasmid construct comprising a green fluorescent protein (GFP) gene, *eGFP*, fused to *AhATL1* under the control of the cauliflower mosaic virus (CaMV) *35S* promoter. The *p35S::AhATL1-eGFP* recombinant gene was transiently expressed in Arabidopsis protoplast cells, resulting in the green fluorescent AhATL-eGFP protein being present at the cell surface, as shown by confocal microscopy. This expression pattern differed from that of GFP alone, which was distributed throughout the cell (**Figure 1C**). These results suggest that *AhATL1* encodes a plasma membrane protein containing conserved ABCG subfamily domains.

### *AhATL1* Responds to Drought Stress via ABA-Specific Signals in Peanut

**FIGURE 2:**
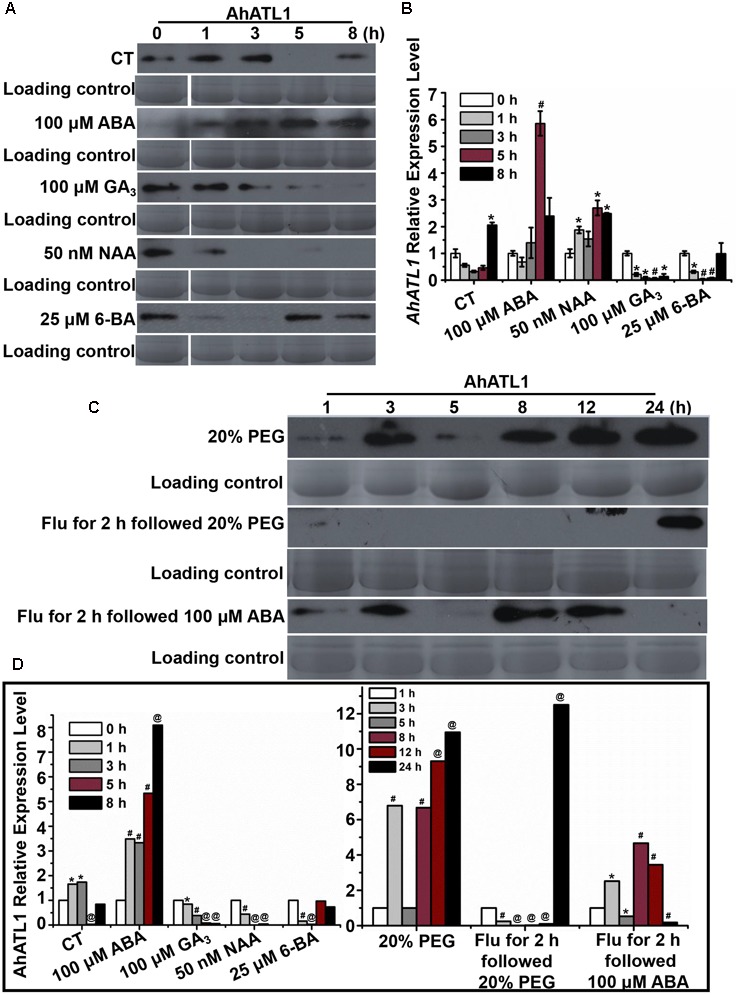
AhATL1 is up-regulated by abscisic acid (ABA) and responds to drought stress in peanut. **(A)** AhATL1 protein accumulation detected by immunoblotting in the first functional leaves of four-leaf peanut plants subjected to 1/8 MS medium (control: CT), 100 μM ABA, 100 μM GA_3_, 50 nM NAA, and 25 μM 6-BA plant hormone treatments and sampled after 0, 1, 3, 5, and 8 h. **(B)** Quantitative PCR of *AhATL1* transcripts in the first functional leaves of four-leaf peanut plants under CT, 100 μM ABA, 100 μM GA_3_, 50 nM NAA, and 25 μM 6-BA plant hormone treatments, sampled after 0, 1, 3, 5, and 8 h. Bars indicate standard deviation (*n* = 3). ‘^∗^’ or ‘#’ indicate a significant difference at the level of *P* < 0.05 or *P* < 0.01, respectively, compared with 0 h under the same treatment. **(C)** AhATL1 protein accumulation detected by immunoblotting in the first functional leaves of four-leaf peanut plants subjected to treatments with 20% polyethylene glycol (PEG), 20% PEG with 100 μM Flu pretreatment or 100 μM ABA with 100 μM Flu pretreatment and sampled after 1, 3, 5, 8, 12, and 24 h. **(D)** Statistical analysis of AhATL1 protein accumulation in **(A,C)** by ANOVA. ‘^∗^’, ‘#’ or ‘@’ indicate a significant difference in protein accumulation at the level of *P* < 0.05, *P* < 0.01 or *P* < 0.001, respectively, compared with 0 or 1 h of the same treatment.

We investigated AhATL1 protein expression in response to treatment of peanut seedlings with the plant hormones ABA, GA_3_, NAA, and 6-BA over a period of 8 h. **Figures 2A,D** shows that AhATL1 protein levels begin to increase after 1 h treatment with 100 μM ABA, reaching a maximum after 5–8 h. Treatment with 100 μM GA_3_ resulted in a gradual decrease in AhATL1 expression, while 50 nM NAA and 25 μM 6-BA also caused a decrease, although with 6-BA this was reversed after 5 h. When we carried out qRT-PCR on total RNA extracted from the same batch of treated seedlings to quantitate levels of *AhATL1* transcripts, we found that gene expression levels were broadly consistent with the immunoblotting results, although GA_3_ treatment did not show a decline in *AhATL1* RNA abundance (**Figure 2B**).

Together, **Figures 2A,B** show that *AhATL1* and its corresponding protein are positively regulated by ABA. Therefore, because ABA is a key phytohormone involved in drought stress (Hwang et al., 2016), we speculated that AhATL1 might be associated with the drought response in peanut. To test this, we simulated water stress by treating peanut roots with 20% PEG 6000 over a period of 24 h, and observed a complicated, non-linear effect on AhATL1 expression, although the overall trend was an increase in protein levels. Thus, at first, the immunoblotting signal increased from 1 to 2 h, decreased between 3 and 5 h, and finally increased continuously from 8 to 24 h (**Figures 2C,D**). To confirm a role for ABA in this induction pattern, peanut roots were pretreated with the ABA synthesis inhibitor Flu (100 μM for 2 h), followed by 20% PEG 6000 treatment, or exogenous ABA (100 μM) was applied alone. Interestingly, no AhATL1 protein signal was detected until 8 h during the 20% PEG 6000 treatment; in contrast, AhATL1 was observed immediately when roots were subject to 100 μM ABA following Flu pretreatment (**Figures 2C,D**). These results demonstrate that *AhATL1* is upregulated by ABA and responds to drought stress via ABA-specific signals in peanut.

### *AhATL1* Reduces Drought Resistance and ABA Sensitivity by Mediating ABA Transport in *AhATL1-OX* Arabidopsis Plants

**FIGURE 3:**
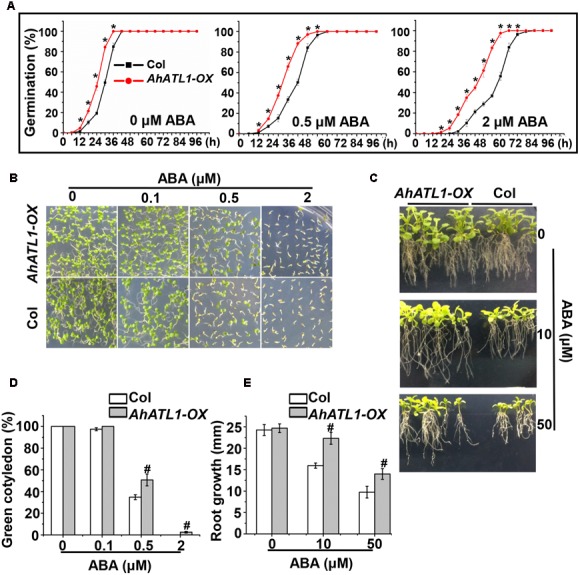
*AhATL1* decreases ABA sensitivity in Arabidopsis. **(A)** Seed germination rate of *AhATL1-OX* and Col lines in response to different concentrations of ABA. Numbers of germinated seedlings were recorded from 0 to 96 h after stratification on 1/2 MS agar plates containing 0, 0.5 or 2 μM ABA. ‘^∗^’ indicates a significant difference at the level of *P* < 0.05. **(B)** Photographs of seedlings grown for 7 days after stratification on agar plates containing 0, 0.1 or 0.5 μM ABA. **(C)** Photographs of seedlings grown for 20 days after transfer to plates containing 0, 10 or 50 μM ABA. **(D)** Proportion of green cotyledons in **(B)**. Bars indicate standard deviation (*n* = 100). ‘#’ indicates a significant difference between *AhATL1-OX* and Col plants under the same ABA treatment conditions (*P* < 0.01). **(E)** Measurement of axial root growth in **(C)**. Bars indicate standard deviation (*n* = 15). ‘#’ indicates a significant difference between *AhATL1-OX* and Col plants under the same ABA treatment conditions (*P* < 0.01).

To understand *AhATL1* function in the context of ABA signaling, we generated transgenic *AhATL1-OX* Arabidopsis lines (i.e., which overexpress *AhATL1*). T3 seedlings from the resultant transgenic lines were tested for ABA sensitivity by measuring germination rate, cotyledon greening and axial root growth. The germination rates of *AhATL1-OX* plants were significantly faster than Col plants when treated with 0.5 or 2 μM ABA, but a difference was even observed without ABA treatment (**Figure 3A**). With respect to cotyledon greening, there was no significant difference between *AhATL1-OX* and Col plants without ABA treatment but, remarkably, *AhATL1-OX* seedlings showed ABA insensitivity at all ABA concentrations tested (0.1, 0.5 or 2 μM; **Figures 3B,D**). Similarly, the axial root growth of *AhATL1-OX* and Col seedlings was identical without ABA, but with ABA treatment root growth of the *AhATL1-OX* seedlings was suppressed to a lesser extent than that of Col seedlings (**Figures 3C,E**). These results indicate that heterologous expression of *AhATL1* reduces ABA sensitivity in Arabidopsis.

**FIGURE 4:**
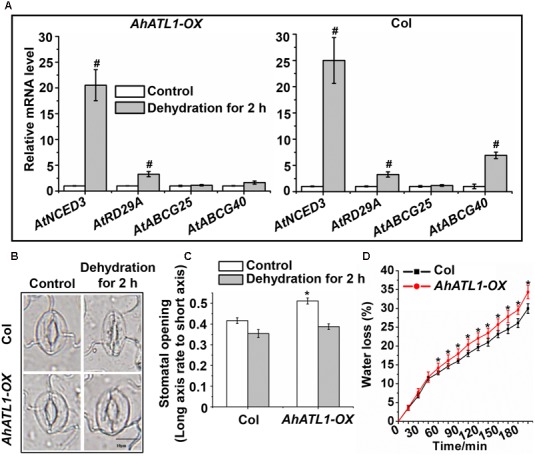
*AhATL1* mediates stomatal movement by reducing expression of ABA import transporter AtABCG40 in Arabidopsis. **(A)** Expression of *AtNCED3*, *AtABCG40*, *AtABCG25*, and *AtRD29A* in *AhATL1-OX* and Col plants under normal or dehydration conditions. Three-week-old *AhATL1-OX* and Col plants were grown normally or under dehydration conditions for 2 h. Bars indicate standard deviation. ‘#’ indicates a significant difference in *AhATL1-OX* or Col plants at the level of *P* < 0.01 between drought stress and control treatment conditions. **(B)** Images (bright-field microscopy) of stomatal apertures in the leaves of 3-week-old *AhATL1-OX* and Col plants under control or dehydration conditions. Bar = 10 μm. **(C)** Stomatal opening in the leaves of 3-week-old *AhATL1-OX* and Col plants under control conditions or after 2 h dehydration; *n* = 150. ‘^∗^’ indicates a significant difference at the level of *P* < 0.05 between *AhATL1-OX* and Col plants under control treatment or drought stress conditions. **(D)** Water loss in the detached leaves of 3-week-old *AhATL1-OX* and Col plants under drought conditions. ‘^∗^’ indicates a significant difference at the level of *P* < 0.05.

We further investigated the effect of *AhATL1* on specific members of the ABA pathway by measuring transcription levels of *AtNCED3*, *AtABCG25*, *AtABCG40*, and *AtRD29A* in *AhATL1-OX* and Col plants subjected to dehydration stress. *AtNCED3* and *AtRD29A* expression was enhanced in both *AhATL1-OX* and Col plants under dehydration conditions, while *AtABCG25* transcription was not affected in either line (**Figure 4A**). A different profile was noted, however, for *AtABCG40*, whose expression was increased in Col plants, but was unchanged in *AhATL1-OX* plants under drought conditions (**Figure 4A**). AtABCG40 is a plasma-membrane ABA-uptake transporter in guard cells, and is necessary for timely closure of stomata in response to drought stress and seed germination (Kang et al., 2010). Therefore, we examined stomatal movement in *AhATL1-OX* and Col plants under water stress. The stomatal aperture in leaves of *AhATL1-OX* plants was larger than Col plants under both normal and dehydration conditions (**Figures 4B,C**). In line with these results, the water loss from detached leaves of *AhATL1-OX* plants was higher than from Col leaves under dehydration conditions (**Figure 4D**). In summary, the above results suggest that *AhATL1* may reduce ABA transport into guard cells, thereby leading to ABA desensitization in Arabidopsis.

## Discussion

The plant hormone ABA regulates various physiological processes, including the responses to stress caused by heavy metals, drought, heat, high levels of salinity, low temperature and radiation (Finkelstein et al., 2002). However, its main function is to regulate plant water balance and osmotic stress tolerance (Finkelstein et al., 2002; Zhu, 2002). The endogenous ABA level is modulated by a precise balance between biosynthesis and catabolism of this hormone (Nambara and Marion-Poll, 2005). The prevailing view has been that, when a plant experiences water stress, the ABA concentration increases in the root due to a local increase in its biosynthesis, and then ABA moves via phloem transport from the roots to the leaves, where it regulates stomatal aperture (Nambara and Marion-Poll, 2005; Rodrigues et al., 2009). More recently, studies in *A. thaliana* report that ABA can also be transported from the leaves to the roots via the phloem when plants are subjected to water limitation or well-watered conditions (Ikegami et al., 2009; McAdam et al., 2016).

Currently, four ABA transporters have been identified in Arabidopsis; these have various transport functions depending on physiological state and tissue (Kang et al., 2010, 2015; Kuromori et al., 2010; Kanno et al., 2012). An understanding of such ABA transporters is essential if we wish to predict the patterns of ABA accumulation at different stress sites and how stomatal apertures respond to water stress. In a previous study in peanut, we found that, when roots are treated with PEG 6000, ABA is synthesized rapidly and accumulates mainly in root vascular parenchyma, after which it is quickly transported to the leaf tissues where it induces stomatal closure. When peanut leaves are treated with PEG 6000, ABA biosynthesis initially increases in the leaf, then rapidly accumulates in the vascular cambium of leaves and induces stomatal closure; ABA produced in root tissues is also transported to the leaf tissues to maintain stomatal closure (Hu et al., 2016). In the current paper, we characterized a novel peanut *ABA transporter-like1* gene, *AhATL1*, which responds to drought stress or exogenous ABA. We found that expression of the *AhATL1* gene and the corresponding protein increased rapidly after treatment with ABA or PEG 6000 (**Figures 2A,B**). However, when roots were pretreated with the ABA synthesis inhibitor Flu, and then subjected to water stress, AhATL1 protein was not detectable (**Figure 2C**). On the other hand, when pretreated with Flu and followed up with ABA treatment, the expression of AhATL1 protein was similar to that observed with PEG 6000 treatment alone (**Figure 2C**). These results prompted us to further explore the role of AhATL1 in ABA signaling.

ABC transporters contain four core structural domains: two of these are TMDs that are located toward the C-terminus of the protein and contain multiple (usually 4-6) membrane-spanning α-helices, which together allow transport of substrates across the lipid bilayer; there are also two NBDs, which are responsible for ATP-binding and hydrolysis (Higgins, 1992; Kerr et al., 2011). In plants, functional ABC transporters consist of a combination of two NBDs and two TMDs organized into a single transporter (full-molecule category), although the core domains may be expressed as separate polypeptides or as multidomain proteins (half-molecule category) (Higgins, 1992). The predicted amino acid sequence shows that AhATL1 is an ABC transporter with TMDs comprising a total of six transmembrane structures, as well as an ABC transporter domain (containing Walker A and Walker B motifs) and ATP-binding sites, consistent with AhATL1 belonging to the family of half-molecule ABC transporters (**Figures 1A,B**). In addition, protoplast transient expression experiments showed that AhATL1 is a plasma membrane protein (**Figure 1C**).

ABA plays a central role in the induction and maintenance of seed dormancy. It also inhibits the transition from embryonic to germination growth (Sah et al., 2016). AtABCG30 and AtABCG40 are ABA-uptake transporters and *Atabcg30* and *Atabcg40* mutant plants are less inhibited by 0.1–2.0 μM ABA than the corresponding Col (wild-type) seeds. *Atabcg40* plants are also defective in stomatal closure, resulting in enhanced water loss and main root growth (Kang et al., 2015). Prompted by these reports, we examined the effects of ABA on the germination and seedling processes on *AhATL1-OX* and Col plants and found that the germination rate of *AhATL1* overexpressing (*AhATL1-OX*) plants is higher than that of Col plants when treated with 0–2.0 μM ABA (**Figure 3A**). There was also more axial root growth and cotyledon greening in *AhATL1-OX* plants, consistent with the enhanced germination rate (**Figures 3B–E**). Hence, these data suggest that overexpression of *AhATL1* decreases ABA sensitivity in Arabidopsis. However, *Atabcg30* and *Atabcg40* mutant plants were inhibited to a lesser degree by ABA than Col seeds, because there is less exogenous ABA transport into plant tissues (Kang et al., 2010, 2015). The key question, then, is why do *AhATL1-OX* plants show a decreased ABA sensitivity phenotype?

Endogenous ABA is rapidly produced during drought, triggering a cascade of physiological responses, including stomatal closure, which is regulated by a signal transduction network. NCED3 in Arabidopsis catalyzes a key step in ABA biosynthesis, and *NCED3* expression is rapidly induced by drought stress. *RD29A* is known as an ABA signaling marker gene due to its rapid response to ABA treatment and drought stress (Shinozaki et al., 2003; Osakabe et al., 2014). Because AhATL1 likely functions as an ABA transporter-like protein in the ABA signaling pathway, we examined the *AtNCED3*, *AtABCG40*, *AtABCG25*, and *AtRD29A* gene transcription levels in *AhATL1-OX* and Col plants under normal and dehydration conditions. The expression of *AtNCED3* and *AtRD29A* was enhanced in both *AhATL1-OX* and Col plants during water stress, but while *AtABCG40* transcription was increased in Col plants, there was no change in expression in *AhATL1-OX* plants. Stomatal activity, which is affected by environmental stress, can influence CO_2_ absorption and water loss, and thus impact photosynthesis and plant growth (Osakabe et al., 2014). When plants experience drought stress, ABA synthesized in roots or leaves is transported into guard cells by specific transporters, such as AtABCG40, which trigger stomatal closure (Hartung et al., 2002; Bauer et al., 2013). Under dehydration stress conditions, we observed reduced stomatal closure in *AhATL1-OX* plants, and, consistent with this, a higher rate of water loss compared to Col plants. This effect is compounded in *AhATL1-OX* plants by the lower levels of *AtABCG40* transcription relative to controls, and the consequently reduced amount of ABA transport into guard cells, causing a further reduction in stomatal movement and higher water loss. Taken together, these observations support the notion that *AhATL1* is involved in ABA transport from synthesis sites to guard cells under dehydration conditions.

In summary, our study reveals for the first time a potential *ABA transporter-like* gene in peanut, which reduces ABA sensitivity by specifically influencing ABA import. This provides insight into the spatial regulation of the ABA transport system and its regulatory networks in *A. hypogaea*.

## Author Contributions

KG, BH, and LL designed the research. KG and XL performed the research. KG, XL, and XyL analyzed the data and prepared figures. KG wrote the manuscript in consulation with BH, XyL, and LL.

## Conflict of Interest Statement

The authors declare that the research was conducted in the absence of any commercial or financial relationships that could be construed as a potential conflict of interest.

## Footnotes

1 https://blast.ncbi.nlm.nih.gov/Blast.cgi

2 http://www.cbs.dtu.dk/services/
